# Characterization of Monochromatic Aberrated Metalenses in Terms of Intensity-Based Moments

**DOI:** 10.3390/nano11071805

**Published:** 2021-07-12

**Authors:** Sorina Iftimie, Ana-Maria Răduţă, Daniela Dragoman

**Affiliations:** 1 Faculty of Physics, University of Bucharest, P.O. Box MG-11, 077125 Bucharest-Magurele, Romania; sorina.iftimie@fizica.unibuc.ro (S.I.); ana.raduta@fizica.unibuc.ro (A.-M.R.); 2 Academy of Romanian Scientists, 3 Ilfov Str., 050044 Bucharest, Romania

**Keywords:** metalenses, aberrations, skewness, kurtosis

## Abstract

Consistent with wave-optics simulations of metasurfaces, aberrations of metalenses should also be described in terms of wave optics and not ray tracing. In this respect, we have shown, through extensive numerical simulations, that intensity-based moments and the associated parameters defined in terms of them (average position, spatial extent, skewness and kurtosis) adequately capture changes in beam shapes induced by aberrations of a metalens with a hyperbolic phase profile. We have studied axial illumination, in which phase-discretization induced aberrations exist, as well as non-axial illumination, when coma could also appear. Our results allow the identification of the parameters most prone to induce changes in the beam shape for metalenses that impart on an incident electromagnetic field a step-like approximation of an ideal phase profile.

## 1. Introduction

Metalenses, constituted from unit cells much smaller than the operating light wavelength, are particularly designed metasurfaces (see, for instance [1,2,3,4,5]) for focusing and imaging purposes. In the last years, metalenses have been thoroughly studied, different configurations of unit cells—meta-atoms—being proposed in order to improve their performances, in particular to minimize their chromatic as well as monochromatic aberrations [6,7]. It was shown that chromatic aberrations can be alleviated by using metalenses doublets [7,8], multilayer metasurfaces [9] or highly anisotropic meta-atoms [10,11] in order to decouple the required phase profile and the material dispersion, or by employing the so-called hybrid metalens design [12], which combines metalenses and phase plates in the same structure. In many applications, however, at least when the available light sources are narrowband, chromatic aberrations can be disregarded; spherical and coma aberrations of metalenses, in particular, becoming significant. These aberrations can be corrected by aplanatic metasurfaces [13] or well-designed doublet metalenses [7]. Note that while an imposed hyperbolic phase profile leads to no spherical aberrations for axially incident fields, an additional phase profile should be added to correct off-axis aberrations—in particular, coma [5,14].

In general, the estimation of aberrations is performed using ray tracing [15,16,17], generalizing the Debye integral and assigning specific aberration types to different Zernike polynomials [18] or assuming a certain expression of the phase distribution imparted by the metalens on an incoming optical field (see [4,19] and the references therein). Irrespective of the aberration type, its effect is to modify the beam shape in the focal plane of the metalens. A quantitative characterization of the spatial distribution of light intensity in terms of a set of numbers is not a trivial task, especially for multi-peaked field distributions. On the contrary, for single-peaked distributions, intensity-defined moments are being used already to characterize the average position, spatial extent and shape of an arbitrary optical field in terms of the first- and second-order moment, and skewness and kurtosis parameters, respectively. The last two parameters, in particular, are employed in order to distinguish the shape of statistical distributions in wide areas of research: in medical imaging for identifying diseases [20,21] and the study of their evolution [22], in material science for characterizing surface corrugations [23], for interpreting data in observational cosmology [24] and altimetry measurements [25], for studying freak waves [26] or winds [27], financial time series [28], or for identifying the topological index in optical vortices [29]. One advantage of using higher-order moments to characterize the shape of an optical beam is that their evolution through first-order optical systems can be readily calculated in terms of the matrices associated to these systems [30].

Whether the skewness, S, and kurtosis, K, are relevant parameters for single-peaked distributions, quantifying the symmetry of a distribution and, respectively, the relative size of tails of a distribution with respect to a normal, Gaussian function, there is no reason to expect that the diffracted field after the metalens is Gaussian-like. On the contrary, as will become clear in the following, the diffracted field is multi-peaked, rippled, but has a dominant maximum on both transverse and longitudinal directions at the focal point. This is the essential feature that allows the use of intensity-based moments, in particular S and K, for characterizing the shape of the diffracted field. As such, the purpose of this paper is to show that, when appropriately defined, in the sense that they mainly capture the information in the dominant peak, these intensity moments can describe the optical field in the focal plane of a metalens and the associated aberrations, and can help with identifying the factors that most affect the focusing performances. This approach is consistent with and generalizes wave-optics simulations of metasurfaces, which should also be used, instead of raytracing, for characterizing aberrations of metalenses. Although restraining our study to monochromatic aberrations only, our analysis is general in the sense that we do not refer to a particular metalens but consider a general diffracting surface consisting of a discrete number of meta-atoms that impart a precisely controllable phase on an incident light field, such that the metalens approximates in a reasonable way the ideal continuous hyperbolic phase distribution of a focusing element. We also assume that the transmission coefficient of the metalens is the same across its surface. Unlike other treatments of aberrations mentioned before, we take into account specifically the discretized phase front after the metalens, which in itself is an important source of aberrations. In all cases studied in this paper the intensity-based moments are used to analyze the influence of different parameters on the field intensity at the focal point. Correlating the results with expected behaviors, we conclude on the appropriateness of using intensity-based moments in characterizing aberrated non-single-peaked distributions.

## 2. Intensity-Defined Moments for Characterizing Metalenses

We study in this paper metalenses under axial and off-axis illumination, conditions under which spherical and coma aberrations, respectively, can occur. Whereas the light intensity distribution of a radially symmetric metalens would keep its symmetry in the presence of spherical aberrations only, such a behavior would no longer be true when coma is present. Therefore, throughout this paper, we assume a radially symmetric metalens, placed in the (*x*,*y*) plane, and follow the intensity distribution through this focusing system in a longitudinal (*x*,*z*) plane (*y* = 0), with *z* being the propagation direction; tilted (with respect to the *x* axis) collimated incident fields make an angle θ≠0 with the *z* axis. Alternatively, we can imagine that the present analysis refers to cylindrical metalenses.

We assume throughout this paper that the metalens is designed to impart a phase profile on an incident light distribution that approximates the ideal, continuous, hyperbolic spatially varying phase distribution *φ_id_*(*x*,*y*) = (2π/λ)[*f* − (*x*^2^ + *y*^2^ + *f*^2^)^1/2^] of a lens with focal length *f* for a given light wavelength *λ*; in our case *y* = 0, so that *φ_id_*(*x*) = (2π/*λ*)[*f* − (*x*^2^ + *f*^2^)^1/2^].

**Figure 1:**
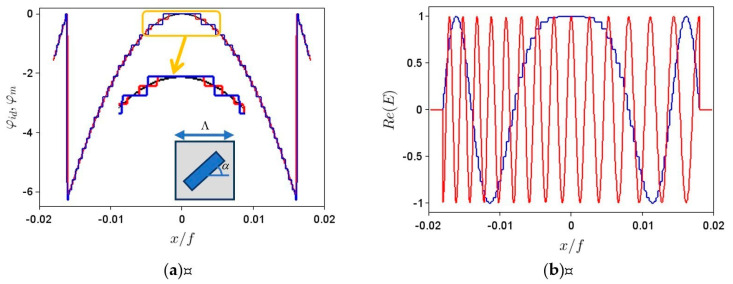
(**a**) Ideal (black line) and step-like approximations when ∆*φ_m_* is 10° (red line) and 20° (blue line) of the phase distribution of a lens with a focal distance of *f* = 10 mm, if *λ* = 1.3 μm, Λ = 450 nm and *N*/2 = 400; part of the figure is enlarged for better visualization of all curves. Inset: unit cell with anisotropic meta-atom at angle α. (**b**) Real part of collimated electric field immediately after the metalens with ∆*φ_m_* = 10° for axial (blue line) and off-axis (red line), at *θ* = 3°, illumination.

The metalenses studied in this paper consist of *N* unit cells with the same transmittance (considered as 1 for simplicity) and the dimension Λ along the *x* axis. The dimension *D* = *N*Λ of the whole metalens is considered as being defined by an opaque aperture, such that no light passes through the metalens plane outside the metalens region. Each cell is supposed to impart a controllable phase *φ_m_* to an incident optical field across its finite dimension. This is the case, for instance, for metalenses formed from anisotropic meta-atoms that can rotate with an azimuthal angle α in the metalens plane (see the inset of Figure 1a); then, for circularly polarized incident fields *φ_m_* = ±2*α* [4,31], the plus and minus signs corresponding to right- and left-circular polarizations, respectively. Thus, because the metasurface is not continuous but is formed from discrete unit cells, it is not possible to generate a continuous phase distribution *φ_id_*(*x*), even if the azimuthal angle α of the metalens varies continuously.

We assume in the following that the azimuthal angle *α* of meta-atoms does not change continuously from one unit cell to the other, but in steps of ∆*α*, the corresponding phase imparted by unit cells being allowed to vary in steps of ∆*φ_m_* = ±2∆*α*. The values of ∆*α* (and thus of ∆*φ_m_*) are chosen by design; for smaller values of this parameter, the ideal phase profile is better reproduced, but the complexity in the fabrication of metalenses is increased. Then, the actual discrete phase distribution imparted by the metalens is

(1) $$\varphi_{m}\left(x\right)=\{\begin{array}{c} \varphi_{step}\left(x\right),\varphi_{step}\left(x\right)-\varphi_{id}\left(x\right)<\Delta \varphi_{m}\left(x\right)/2\\ \varphi_{step}\left(x\right)-\Delta \varphi_{m},\varphi_{step}\left(x\right)-\varphi_{id}\left(x\right)\geq \;\Delta \varphi_{m}/2 \end{array}$$

where *φ_step_*(*x*) = ∆*φ_m_*Int[*φ_id_*(*x*)/∆*φ_m_*], with Int[] denoting the integer part of the argument. This discretization of the phase profile leads to unavoidable errors in phase implementation. The normalized distance between the ideal and step-like phase profiles could be estimated as $\sigma =\sqrt{\underset{x_{j}}{\sum }{\left[\varphi_{id}\left(x_{j}\right)-\varphi_{m}\left(x_{j}\right)\right]}^{2}/\underset{x_{j}}{\sum }\varphi_{id}^{2}\left(x_{j}\right)}$, where the sum is taken over the centers *x_j_* = Λ/2 + (*j* − 1)Λ of all *N* unit cells that form the metalens, with *j* an integer such that −*N*/2 < *j* ≤ *N*/2.

For example, Figure 1a illustrates (modulo 2*π*) the ideal phase distribution of a lens with a focal distance of *f* = 10 mm (black line), as well as the step-like approximations when ∆*φ_m_* is 10° (red line) and 20° (blue line) if *λ* = 1.3 μm, Λ = 450 nm and *N*/2 = 400; these values for *f*, *λ* and Λ will be used throughout the paper. The obtained *σ* values are about 2.2% for the metalens with a 20° step in ∆*φ_m_* and of only 1.1% for that with a 10° step; although these values are not high, the changes in beam shape can be significant. The inset of Figure 1a represents a unit cell of the metalens with a rotated meta-atom.

**Figure 2:**
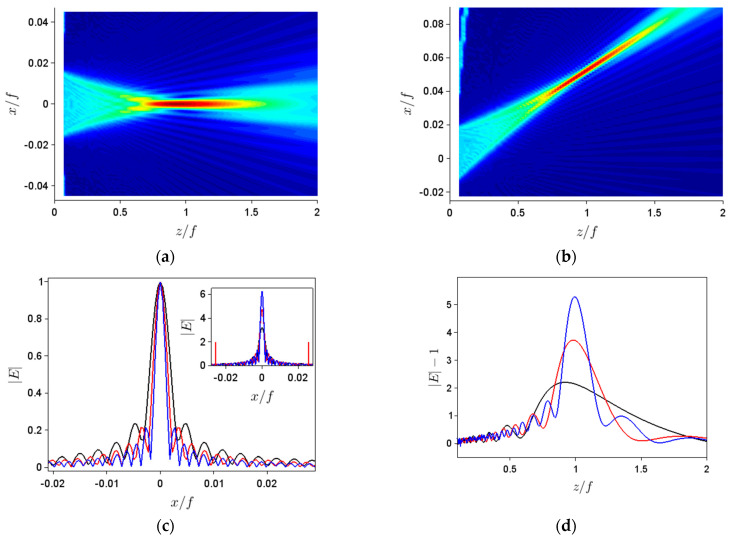
(**a**,**b**) Spatial dependences of the absolute values of diffracted electric fields in Figure 1b. Spatial distributions in the (**c**) transverse and (**d**) longitudinal planes of the absolute value of diffracted electric fields for ∆*φ_m_* = 10°, *f* = 10 mm, and *N*/2 = 400 (black line), 600 (red line) and 800 (blue line). (**c**) Normalized field distributions; inset: not normalized. The vertical red lines in the inset show the extent of the region taken into account for higher-order moments calculation for *N*/2 = 600.

We assume in the following that the metalens is illuminated by a collimated optical field given by *E_inc_*(*x*) = *E*_0_exp(*i*2*πθx*/*λ*), tilted at an angle *θ* and normalized such that *E*_0_ = 1, the field immediately after the metalens surface, situated at *z* = 0, being *E*(*x*, *z* = 0) = *E_inc_*(*x*)exp[*iφ_m_*(*x*)]. The real part of the field transmitted by the metalens in Figure 1a with ∆*φ_m_* = 10° is shown in Figure 1b with a blue line for normal incidence, *θ* = 0, and with a red line for tilted incidence, with *θ* = 3°. The spatial distributions of the absolute value of the electric fields for the normal and tilted incidence cases in Figure 1b, after the metalens, are illustrated in Figure 2a and, respectively, Figure 2b. All spatial coordinates are normalized to the focal length *f* = 10 mm of the metalens. All simulations have been carried out with the open-source program Scilab.

Assuming that *N* is an even number, we have calculated the electric field profile for *z* > 0 applying the diffraction integral in the Fresnel approximation [32] (our working regime is near-field) to the one-dimensional discontinuous optical field *E*(*x*, *z* = 0):

(2) $$E\left(x,z\right)=\frac{1}{\sqrt{i\lambda z}}\exp\left(ikz\right)\int_{-N\Lambda /2}^{N\Lambda /2}E\left(x^{\prime },z=0\right)\exp\left[\frac{ik}{2z}{\left(x-x^{\prime }\right)}^{2}\right]dx^{\prime }$$

To pinpoint the focal plane position along the *z* axis, as well as for describing the transverse field distribution along the *x* axis, we need to look at the field and intensity *I* ≈ |*E*|^2^ distributions along both *x* and *y* axes; thus, we have extracted the following parameters:

- the average position of the intensity distribution along the transverse *x* and longitudinal *z* axes, defined via the first-order moment of the intensity *I*(*x*,*z*) as:
  (3) $$\xi_{av}=\langle \xi \rangle =\int^{\;}\xi I\left(\xi \right)d\xi /\int^{\;}I\left(\xi \right)d\xi ,\;\;\xi =x,z$$
- the spatial extent of the intensity distribution along the *x* and *z* axes, defined via the second-order moment of the intensity as [33]
  (4) $$\Delta \xi =4\sqrt{\langle \xi^{2}\rangle }=4\sqrt{{\left(\xi -\xi_{av}\right)}^{2}I\left(\xi \right)d\xi /\int^{\;}I\left(\xi \right)d\xi },\;\;\xi =x,z$$
- the shape of the intensity distribution along the *x* and *z* axes, parameterized via the skewness *S* and kurtosis *K* coefficients defined as:
  (5) $$S_{\xi }=\langle \xi^{3}\rangle /\langle \xi^{2}\rangle {}^{3/2},\;\;\;K_{\xi }=\langle \xi^{4}\rangle /\langle \xi^{2}\rangle {}^{2},\;\;\xi =x,z$$

with $\langle \xi^{m}\rangle =\int^{\;}{\left(\xi -\xi_{av}\right)}^{m}I\left(\xi \right)d\xi /\int^{\;}I\left(\xi \right)d\xi$, *m* = 3, 4. As mentioned before, the skewness quantifies the (lack of) symmetry of a distribution, negative (positive) *S* values indicating distributions that have a left (right) tail longer than the right (left) tail for single-peaked distributions, while *S* = 0 corresponds to a distribution that is symmetric to the left and right. The kurtosis, *K*, on the other hand, evaluates the relative size of tails of a single-peaked distribution with respect to a normal, Gaussian function, for which *K* = 3 (and S = 0). Thus, *K* values higher (lower) than 3 indicate distributions that have heavy (light) tails with respect to a Gaussian.

In our case, however, the field distributions of interest are not single-peaked. For axial illumination, for instance, we are interested in the spatial field distribution along the *z* axis (for *x* = 0), as well as for the transverse field distribution at the focal point; the latter was defined as the *x*-distribution at the *z* coordinate at which the absolute value of the *z*-dependent field is the maximum. These distributions, shown in Figure 2c,d, reveal that the field after the metalens, although displaying a dominant maximum near the focal point of the perfect lens, shows a multi-modal structure along both *x* and *z* axes, with pronounced ripples along *x*. These ripples especially affect the *S* and *K* values (and not so much the average positions and spatial extents), especially since their maxima vary with different parameters used in simulations. As a result, in order to obtain intensity-based moments pertaining only to the main peak of the field distribution along *x*, we have first determined *x*_av_ and ∆*x*, taking into account absolute values of the electric field higher than 1/40 of the peak value, and then calculated *S* and *K*, taking into account only the electric fields with absolute values in an interval of 5∆*x* centered around *x*_av_. As can be seen from the inset of Figure 2d, where the relevant region for *N*/2 = 600 (the red curve) has been highlighted by the two (also) red vertical lines, this interval is broad enough to account for the tail of the field intensity. No such precautions were needed for calculating the intensity-based moments along the *z* axis, except for considering an electric field with higher absolute values than the background (equal to 1), which was removed.

## 3. Numerical Simulations Results

### 3.1. Axial Illumination of Metalenses

**Figure 3:**
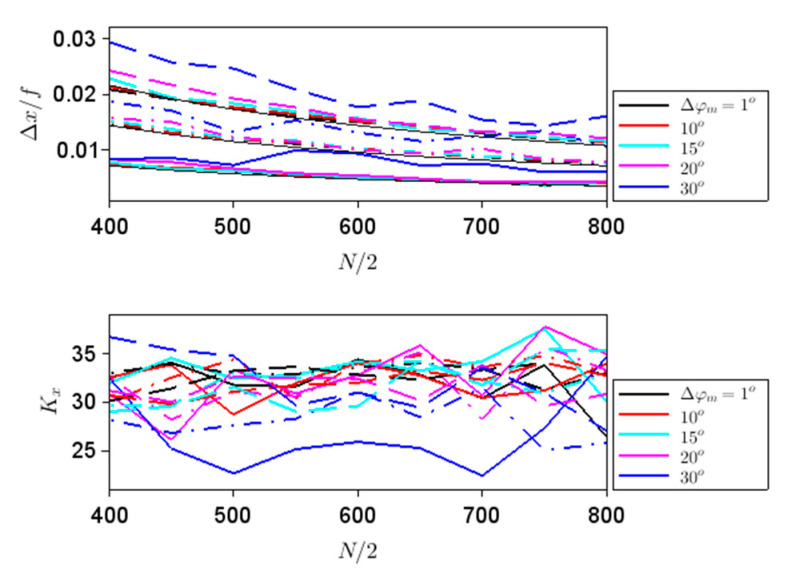
∆*x* (top) and *K_x_* (bottom) dependences on aperture size for different steps in ∆*φ_m_* indicated in the legend and focal lengths of *f*/2 (solid line), *f* (dashed–dotted line) and 1.5*f* (dashed line). The thin black lines in the top figure represent the dependences on *N* of the function 2*λ*/*N* Λ = *λ*/*NA*.

**Figure 4:**
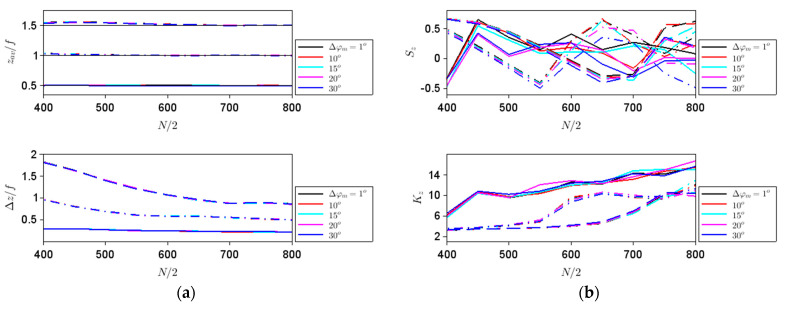
Dependences on the aperture size of (**a**) *z_av_* and ∆*z*, and (**b**) *S_z_* and *K_z_* for different steps in ∆*φ_m_* indicated in the legend and focal lengths of *f*/2 (solid line), *f* (dashed–dotted line) and 1.5*f* (dashed line). The thin black lines in the top figure (**a**) represent the geometric location of the focal spot.

In this case, discretization-induced focusing errors are dominant since spherical aberrations are not expected to be present for hyperbolic metalenses, at least from a wave-optics point of view. We have considered metalenses with different apertures, namely with *N*/2 = 400 to 800 in steps of 50, different focal lengths: *f*/2, *f* and 3*f*/2, with *f* = 10 mm, as well as different steps ∆*φ_m_*: 1° (almost continuous rotation of meta-atoms), 10°, 15°, 20° and 30°. The calculated parameters defined in terms of intensity-based moments along *x* and *z* are represented in Figure 3 and Figure 4, respectively; due to the symmetry of the problem *x_av_* = 0 and *S_x_* = 0 in all cases. In these figures, as well as in the following ones, the steps in ∆*φ_m_* are indicated in the legend and the corresponding curves are plotted with different colors, while the curves corresponding to *f*/2, *f* and 3*f*/2 are drawn with different line types: continuous, dashed–dotted and dashed lines, respectively. The results in Figure 3 show that the spatial extent of the focal point along the *x* axis increases with *f*, while the evolution of ∆*x* with the aperture size is in agreement with the simulations in Figure 2c. The three thin black lines in the upper Figure 3 represent the dependencies on *N* of the function 2*λ*/*N*Λ = *λ*/*NA*, i.e., of the double of the Abbe diffraction limit; it can be seen that ∆*x* defined in terms of second-order intensity moments is related to this measure of the diffraction limit and almost coincides with it except for large phase discretization steps.

Indeed, for phase steps of 30°, the focal spot increases in magnitude, except for small *f* values and low diameters. On the other hand, the overall shape of the light distribution in the focus, parameterized by *K_x_*, does not depend very much on the phase step and the size of the aperture, unless the phase discretization step is large; in this last case, the beam shape becomes less heavy-tailed. Both ∆*x* and *K_x_* are good indicators of the expected behavior that the focusing performances deteriorate with an increase in ∆*φ_m_*.

Along the *z* direction, all parameters defined above have a very weak dependence on the phase step, as can be seen from Figure 4. The average position of the intensity maximum is in all cases close to the geometrical focus, depicted with thin black lines in the upper Figure 4a, and gets closer to this geometric value as the aperture size increases, while the spatial extent of the focal spot decreases with increasing *N*, as well as with the increase of the focal strength.

**Figure 5:**
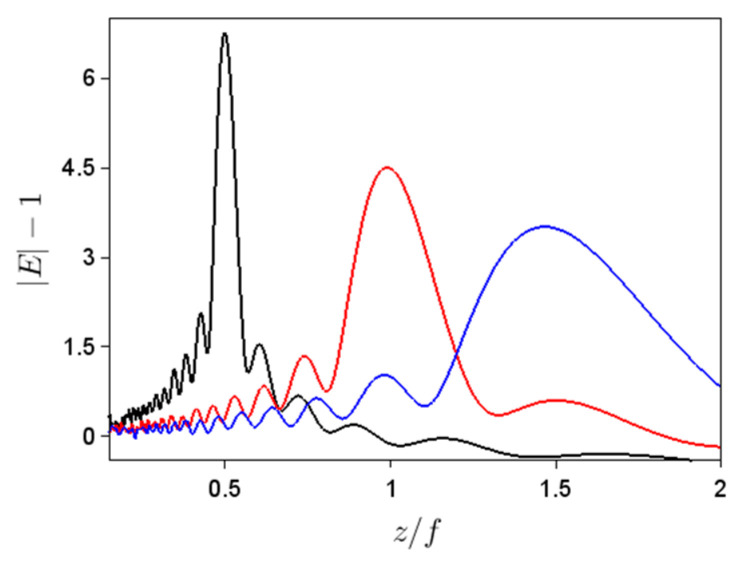
Dependence on *z* of the modulus of the electric field for ∆*φ_m_* = 10°, *N*/2 = 700, and focal length *f*/2 (black line), *f* (red line) and 1.5*f* = (blue line).

While these behaviors could be expected from general considerations, the strong variations of *S_z_* and *K_z_* with both *N* and *f* suggest that the shape of the focalized beam depends on these parameters, in agreement with the simulations in Figure 2d, performed for different *N* values, and Figure 5, performed for different *f* values. Thus, both *S_z_* and *K_z_* are good indicators of the beam shape variation along the *z* axis.

### 3.2. Non-Axial Illumination of Metalenses

**Figure 6:**
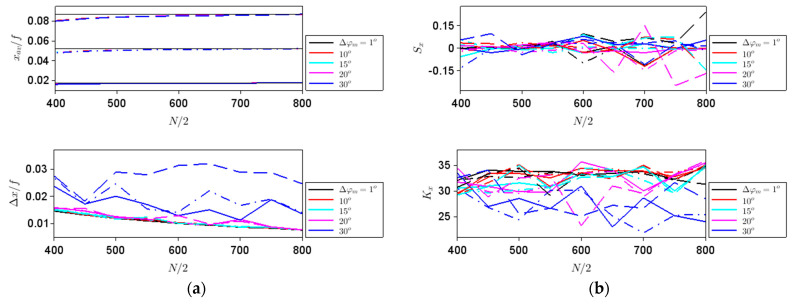
Dependence on the aperture size of (**a**) the transverse average position and spatial extent of the focalized beam and (**b**) of *S_x_* and *K_x_* for different phase steps indicated in the legend and for *θ* = 1° (solid line), 3° (dashed–dotted line) and 5° (dashed line).

**Figure 7:**
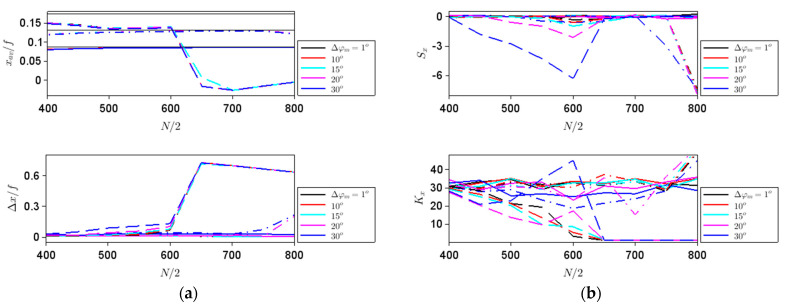
Dependence on the aperture size of (**a**) the transverse average position and spatial extent of the focalized beam and (**b**) of *S_x_* and *K_x_* for different phase steps indicated in the legend and for *θ* = 5° (solid line), 7.5° (dashed–dotted line) and 10° (dashed line).

**Figure 8:**
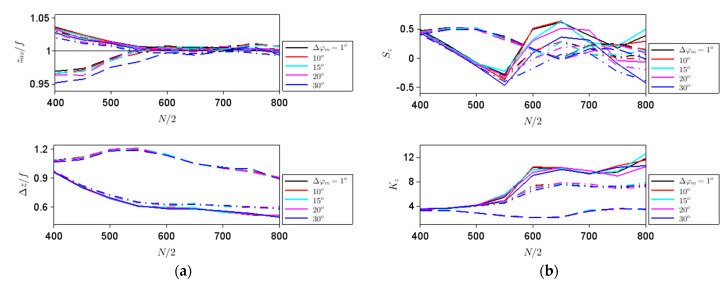
Dependence on the aperture size of (**a**) the transverse longitudinal position and spatial extent of the focalized beam and (**b**) of *S_z_* and *K_z_* for different phase steps indicated in the legend and for *θ* = 5° (solid line), 7.5° (dashed–dotted line) and 10° (dashed line).

In this case, the coma aberration is expected to appear, besides the discretization-induced aberrations. As in the previous case, we have plotted the parameters defined in terms of intensity moments for different phase discretization steps and aperture sizes. As shown in Figure 2b, the light field propagates in this case along a tilted direction, to find the relevant *x*- and *z*-dependent field distributions and the corresponding intensity-based moments; consequently, we have first plotted the field along the propagation direction (along *x* = *z*tan*θ*) and chosen the transverse *z* = const. focusing plane as that for which the intensity is the maximum. Figure 6a plots the dependence on the aperture size of the transverse average position and spatial extent of the focalized beam for different phase steps indicated in the legend and for three tilt angles: *θ* = 1° (solid line), 3° (dashed–dotted line) and 5° (dashed line), while Figure 6b represents, with matching line types, the corresponding dependences of *S_x_* and *K_x_*. The thin black lines in the upper Figure 6a indicate the geometrical positions *x* = *f*tan*θ*. As can be seen from these figures, except for ∆*φ_m_* = 30°, and for ∆*φ_m_* = 20° at large apertures, the beam shapes do not vary significantly; the transverse average position is close to the geometric location, and becomes closer as the aperture size increases, while the spatial extent of the beams decreases as *N* increases, as in the case of axial illumination. However, as the tilting angle increases below 7.5°, the behavior of all intensity-based moments changes dramatically. This can be seen from Figure 7, which represents the same parameters as Figure 6, with similar line types but for *θ* = 5° (solid line), 7.5° (dashed–dotted line) and 10° (dashed line), and also from Figure 8, which illustrates with the same line types as Figure 7 the aperture size dependence of the corresponding parameters along the *z* direction (basically, along the tilted propagation direction). In the last case, the dependences for *θ* = 1°, 3° and 5° almost superimpose each other (as for the axial illumination case), so we have not represented these cases separately.

**Figure 9:**
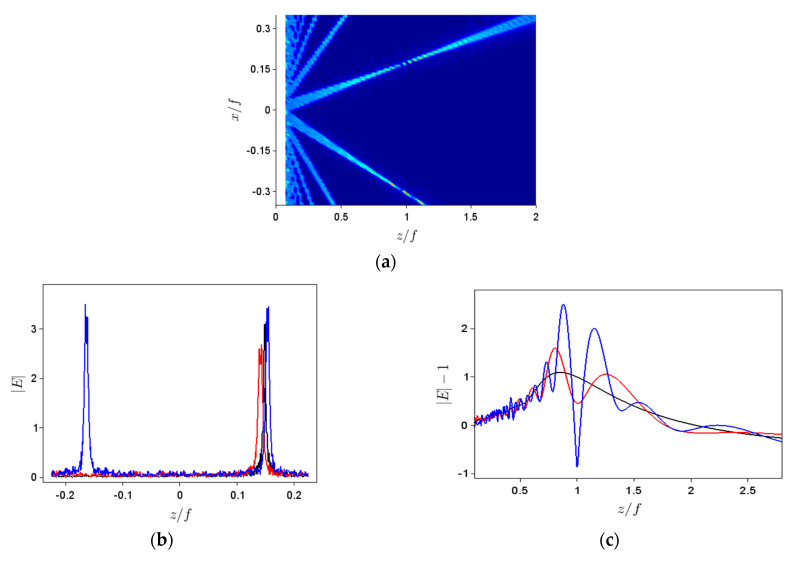
(**a**) Spatial distribution of the field modulus for *θ* = 10°, *N*/2 = 600 and ∆*φ_m_* = 30°. Corresponding (**b**) transversal and (**c**) longitudinal field distributions for *N*/2 = 400 (black line), 600 (red line) and 800 (blue line).

To understand why the behavior of all intensity-based moments at large tilting angles changes dramatically—change that is more pronounced for larger aperture sizes—we have plotted the spatial distribution of the absolute field values in this case (see Figure 9a) and have discovered that, besides the propagation direction imposed by the tilting angle, constructive interferences appear along other directions, such that we no longer have one propagating beam but several. Figure 9b,c show the transversal and longitudinal, respectively, field distributions in this case for ∆*φ_m_* = 30° and *N*/2 = 400 (black line), 600 (red line) and 800 (blue line). A second transverse peak is clearly visible in this case for the largest aperture size. The *z*-distribution of the field no longer has one obvious maximum for larger apertures, but several, in agreement with the simulations in Figure 9a. Again, the change in beam shape, especially significant at high tilt angles and high apertures, is well-described by *S_x_*, *K_x_*, *S_z_* and *K_z_* parameters, whereas the appearance of additional beams propagating at other angles has a pronounced effect also on the spatial extent and average position values along both *x* and *z*.

## 4. Discussions and Conclusions

We have shown, through extensive numerical simulations, that intensity-based moments and the associated parameters defined in terms of them (average position, spatial extent, skewness and kurtosis) adequately capture changes in beam shapes induced by aberrations. The approach towards investigating aberrations taken in this paper is novel in the study of metalenses, for which ray-optics and the associated phase distribution-based methods are the techniques of choice [15,16,17,18,19]. On the other hand, our approach is consistent with the general numerical simulation techniques of metalenses, which rely on wave optics. In addition, for subwavelength structures with discretized phase profiles, which are the hallmark of metalenses, it is debatable if ray optics is an appropriate tool.

As we have taken as a working example a metalens with a hyperbolic phase profile, only phase-discretization-induced aberrations (not studied in other works) could have occurred for axial illumination, while coma could have been present at non-axial illumination. As our approach can take into account tilts in different planes and aperture dimensions, parameters that can be associated with different standard types of aberrations, and even different incident beam shapes, our focus was to identify the parameters most prone to induce changes in the beam shape.

In particular, for axial illumination, we have found that the most detrimental parameter is the phase discretization step: values of ∆*φ_m_* higher than 20° affect the focal spot along the *x* direction, but do not influence the field distribution along *z* in the case of axial illumination, the focal length inducing significant changes in this distribution together with the aperture size. In general, higher aperture sizes lead to better field focalization along both transverse and longitudinal directions, and to an increase in the tails of the field distribution. Again, phase steps higher than 20° considerably influence the spatial extent along *x* and the tails of the field distribution along this axis for non-axial illumination, having basically no effect on the field distribution along *z*. However, all intensity-based moments change dramatically at large tilting angles, at which additional paths of constructive interference form. The change is much more significant at large apertures, meaning that larger apertures favor the appearance of new constructive interference paths. All these results are not unexpected; they do not in themselves state something new but confirm the fact that metalenses aberrations can be described using wave-optics, in particular using intensity-based moments of light distributions. These results also show that phase discretization steps should be minimized in proper designed metalenses, while the diameters of the metalenses should be limited if non-axial illumination is envisaged.

These conclusions, although drawn following simulations of a certain metalens (with well-defined parameters), are believed to be generally due to scaling laws in wave optics. The results in this paper show that, even along both *x* and *z* axes, the spatial distribution of a diffracted field is not single-peaked; the *S* and *K* parameters are appropriate for describing the shape of the light distributions, and as such generalizes the research fields in which these parameters can be used. However, when significant ripples or, in general, noise is present, one has to be careful of the way to calculate intensity-based moments: higher-order moments are more prone to errors. This is the reason why we have defined a certain range for relevant field values only for the *x* direction. The extent of this range depends on the field distribution itself but must be chosen attentively before any analysis of field shape can be performed.

## Data Availability

The authors confirm that the data supporting the findings of this study were generated using the method, software and parameters indicated within the article.
